# Assessment of Prosthesis Alignment after Revision Total Knee Arthroplasty Using EOS 2D and 3D Imaging: A Reliability Study

**DOI:** 10.1371/journal.pone.0104613

**Published:** 2014-09-23

**Authors:** Marrigje F. Meijer, Alexander L. Boerboom, Martin Stevens, Sjoerd K. Bulstra, Inge H. F. Reininga

**Affiliations:** 1 Department of Orthopaedics, University of Groningen, University Medical Center Groningen, Gronigen, The Netherlands; 2 Department of Trauma Surgery, University of Groningen, University Medical Center Groningen, Groningen, The Netherlands; Steno Diabetes Center, Denmark

## Abstract

**Introduction:**

A new low-dose X-ray device, called EOS, has been introduced for determining lower-limb alignment in 2D and 3D. Reliability has not yet been assessed when using EOS on lower limbs containing a knee prosthesis. Therefore purpose of this study was to determine intraobserver and interobserver reliability of EOS 2D and 3D knee prosthesis alignment measurements after revision total knee arthroplasty (rTKA).

**Methods:**

Forty anteroposterior and lateral images of 37 rTKA patients were included. Two observers independently performed measurements on these images twice. Varus/valgus angles were measured in 2D (VV2D) and 3D (VV3D). Intraclass correlation coefficients and the Bland and Altman method were used to determine reliability. T-tests were used to test potential differences.

**Results:**

Intraobserver and interobserver reliability were excellent for VV2D and VV3D. No significant difference or bias between the first and second measurements or the two observers was found. A significant mean and absolute difference of respectively 1.00° and 1.61° existed between 2D and 3D measurements.

**Conclusions:**

EOS provides reliable varus/valgus measurements in 2D and 3D for the alignment of the knee joint with a knee prosthesis. However, significant differences exist between varus/valgus measurements in 2D and 3D.

## Introduction

Achieving optimal prosthetic alignment during total knee arthroplasty (TKA) is an essential part of the surgical procedure. Malpositioning of a knee prosthesis in the coronal plane causes earlier loosening and revision surgery [1]. Revision TKA (rTKA) has to be prevented, as this is associated with worse functional outcome and prosthesis survival [2], [3]. Proper alignment in the coronal plane is associated with less pain, better knee function, faster rehabilitation and improved quality of life [4], [5]. Optimal coronal alignment is considered ≤3° varus or valgus [6].

Conventional weight-bearing radiographs are generally used to measure alignment in the coronal and sagittal planes. Proportions and angles may not be correct though, given the divergence in the vertical and horizontal planes. A computed tomography (CT) scanogram can also be used to evaluate prosthetic alignment in the coronal, sagittal and rotational planes. However, due to high levels of radiation and high costs it cannot be used routinely. Moreover, with a CT-scan it is not possible to obtain images of the leg in weight-bearing position.

The EOS system has been developed for the evaluation of prosthetic alignment (EOS Imaging, Paris, France) [7]. With this biplanar low-dose X-ray technique, orthogonally made long-leg 2D radiographs and 3D reconstructions can be obtained. Major advantages are that images of the leg are obtained on a 1∶1 scale with an amount of radiation 800–1000 times lower than CT-scans and 10 times lower than conventional X-rays [7], [8]. However, the EOS software for creating 3D reconstructions is developed for lower limbs without knee prosthetic material. When a knee prosthesis is *in situ*, several anatomical reference points have disappeared or changed, making it difficult to mark reference points as described by the measurement protocol. Therefore, the measurement protocol was adjusted. Reliability of this protocol have not been investigated yet.

Purpose of this study was to determine intraobserver and interobserver reliability of 2D and 3D knee prosthesis alignment measurements after rTKA using EOS. As a secondary outcome we assessed whether significant differences existed between 2D and 3D measurements.

## Materials and Methods

Fifty-four patients who underwent rTKA between January 1998 and November 2009 and who were available for the acquisition of EOS images between November 2009 and May 2010 were included. An anteroposterior (AP) and lateral (LAT) image of the operated leg was made with the EOS stereography system at the Radiology Department of our hospital as part of the standard follow-up protocol for rTKA. In accordance to regulations of the Medical Ethical Review Board of University Medical Center Groningen, patients were informed about the fact that data of their radiographs could be used for scientific research. If patients had objections to the use of their data these data were not included in the study.

Patients were positioned on the EOS platform in standing position with the right foot 10 cm in front of the left foot. SterEOS software (Biospace Imaging, Paris) was used to take 2D measurements of the AP images and 3D measurements of the AP and LAT images. The images were anonymized by removing names, patient numbers and birth dates. The guidelines for taking measurements as provided by the manufacturer were followed [9]. Since several landmarks disappear or change when a knee prosthesis is *in situ*, the observers made the following agreements on marking the landmarks:

– Instead of the center of tibial spines, the center of the tibial  plateau is chosen;
– Instead of marking the distal femoral notch, the center of the  femoral component is marked;
– Instead of marking the anatomic femoral condyles, the  condyles of the femoral component are marked.

**Figure 1:**
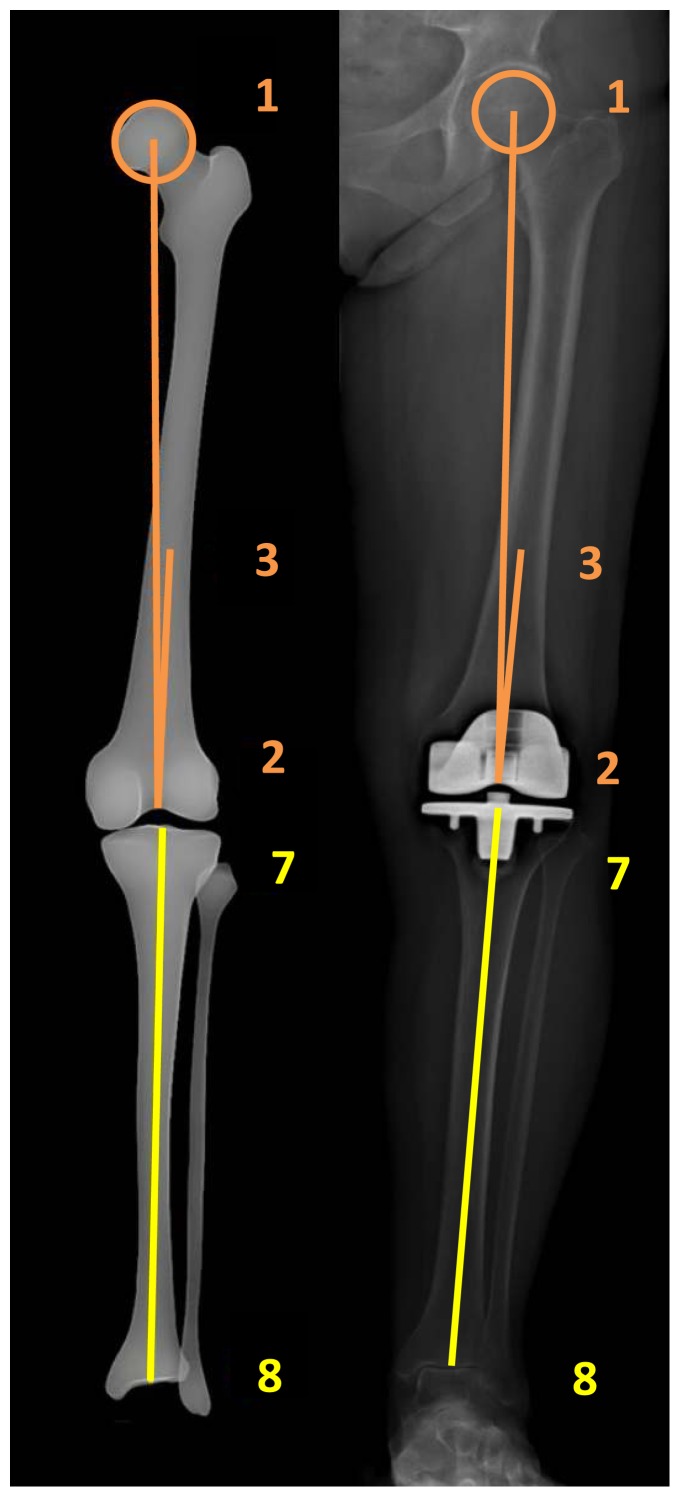
Identification of the lower limb on the frontal image.

**Figure 2:**
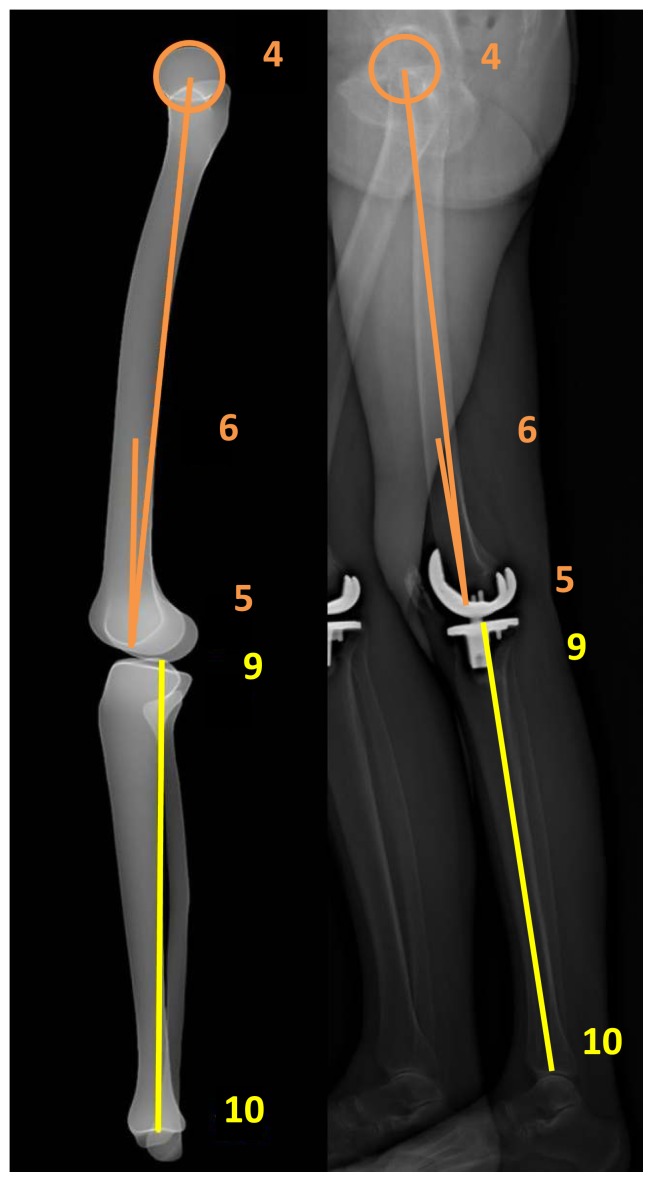
Identification of the lower limb on the lateral image.

In order to calculate coronal and sagittal alignment parameters of the lower limb in 2D and 3D, the “lower limb alignment” mode is used. The first step is to define the left or right lower limb and to choose the modeling “lower limb alignment” mode. Next, identification of the lower limb on the AP and LAT images is done in 10 steps (Figure 1 and Figure 2):

Femur:

- Center of femoral head (points 1 and 4);
- Center of the distal femoral notch (points 2 and 5);
- Center of the diaphysis in its distal third (points 3 and 6).

Tibia:

- Center of the tibial spines. When a knee prosthesis is in situ  the tibial spines disappear, therefore the center of the tibial  plateau is chosen and the axis from the center of the ankle to  the center of the tibial plateau represents the anatomical axis  of the tibia (points 7 and 9);
- Center of the distal articular surface in the upper ankle joint  (points 8 and 10).

**Figure 3:**
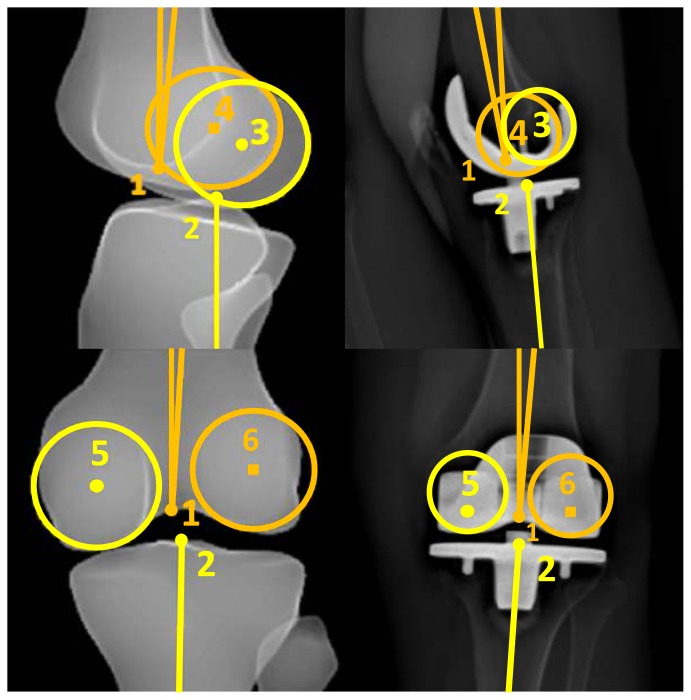
Adjustment of the landmarks on the frontal and lateral images.

The next step is adjustment of the landmarks in four steps (Figure 3):

1. Adjustment of the position of the sphere of the femoral head in both views. It is possible to enlarge or minimize the size of the sphere according to the size and shape of the femoral head, in order to mark the center of the femoral head as precisely as possible;
2. Adjustment of the point in the center of the distal third of the diaphysis of the femur;
3. Adjustment of the position of the point in the center of the femoral notch and tibial plateau, and marking of the femoral condyles. The condyles have to be identified on the AP and LAT images using the two spheres. It is possible to adjust the size of the spheres, according to the size of the condyles. On the AP image the center of the spheres has to be located in the center of each condyle. On the LAT image the spheres have to be tangent to the posterior part of the condyles. It is important not to confuse the medial with the lateral condyles. In order to identify the right condyle, the epipolar line is used to differentiate between the two condyles by observing the correspondence of condylar height on both the AP and the LAT image;
4. Adjustment of the reference point in the center of the distal articular surface on the AP and LAT images.

VV2D is the angle between the mechanical axis of the femur (axis between points 1 and 2) and the tibia (axis between points 7 and 8) on the AP image (Figure 1). For the 3D measurement, the points marked on the AP (Figure 1) and LAT (Figure 2) images as described above are combined to generate the mechanical axes of femur and tibia. VV3D is the angle between the three-dimensional mechanical axis of the femur (axis between points 1–4 and 2–5) and tibia (axis between point 7–9 and 8–10).

Primary outcome measurement is the varus/valgus angle (VV) (angle between the mechanical axes of femur and tibia) in 2D (VV2D) and 3D (VV3D) because of its clinical importance. A positive value indicates valgus and a negative value indicates varus.

An independent researcher randomly numbered all images twice. In this way, two blinded sets of 40 AP and LAT images each were composed. Two observers (observer A and observer B) separately analyzed both sets of 40 images twice. Both observers were experienced in taking the measurements in 2D and 3D prior to the study.

### Statistical analyses

Statistical analyses were performed using the PASW software package (version 18, SPSS, Chicago). Intraobserver and interobserver reliability were investigated by determining relative and absolute reliability [10]. Relative and absolute intraobserver reliability were investigated by respectively calculating intraclass correlation coefficients (ICCs) and using the Bland & Altman method [10]. The ICCs with 95% confidence interval (CI) for each 2D and 3D measurement were calculated and interpreted according to the benchmarks described by Fleiss. An ICC >0.75 represents an excellent correlation, 0.40–0.75 a moderate-to-good correlation and <0.40 represents a poor correlation [11].

Absolute intraobserver and interobserver reliability were calculated by the Bland & Altman method [12]. For intraobserver reliability the mean difference and 95% CI between measurement set 1 (M1) and measurement set 2 (M2) were calculated for both observers separately. For interobserver reliability the mean difference and 95% CI between the two observers were calculated. When intraobserver reliability was good for both observers, the means of M1 and M2 of observer A (n = 40) were compared with the means of both sets of observer B (n = 40).

To investigate agreement on the number of outliers between M1 and M2, as well as the two observers, Cohen's κ coefficients were calculated [13]. Angles with a deviation >3° varus or valgus from the neutral axis were considered outliers [6]. The κ values were interpreted according to Landis and Koch [14]: <0 represents less than chance agreement, 0.01–0.20 represents slight agreement, 0.21–0.40 fair agreement, 0.41–0.60 moderate agreement, 0.61–0.80 substantial agreement and 0.81–0.99 represents almost perfect agreement. χ^2^ tests were performed to assess statistically significant differences in the number of outliers.

To identify significant differences between M1 and M2, a paired Student T-test was performed and the standard error of measurement (SEM) and smallest detectable change (SDC) were calculated. The formulas used to calculate the SEM and SDC are respectively SEM  =  standard error of difference/√2 and SDC  = 1.96×√2× SEM. [15]–[17] A Student T-test for independent samples was performed to assess significant differences between the means of the measurements of the two observers, and the SEM and SDC were calculated.

Potential differences between VV2D and VV3D measurements were assessed using T-tests. First, the means of M1 and M2 of each observer for both VV2D and VV3D were calculated, creating a VV2D and VV3D set (n = 40/n = 40) for each observer. Next, the means of the mean of observer A and observer B for both VV2D and VV3D were calculated. In this way, one set of VV2D and one set of VV3D measurements was generated (n = 40/n = 40). A Paired-samples T-test was performed to detect any significant differences between both sets. The absolute difference between VV2D and VV3D was calculated for each subject. Meaning, the deviation of the neutral axis was stated as a positive value, regardless of the deviation being varus or valgus. The absolute differences were compared with the value 0 using a One-sample T-test, since a zero value indicates no absolute difference between VV2D and VV3D. Additionally, Cohen's κ was calculated to determine agreement on the number of outliers between measurements of VV2D and VV3D, with an outlier defined as >3° varus or valgus. A χ^2^ test was performed to assess statistically significant differences in the number of outliers between VV2D and VV3D. For all statistical analyses, a p-value of <0.05 was considered to indicate statistical significance.

## Results

On 14 of the 54 images it was not possible to identify medial and lateral condyles of the femoral component on the LAT X-ray and were excluded from further analysis. Eventually, 40 AP and LAT images were available for final analysis. The patient population consisted of 21 men and 16 women, with a mean age of 64.5 years (range 32–83). Of the 40 sets of images, 23 images were made of the left lower limb and 17 of the right lower limb.

Relative intraobserver reliability was excellent when measuring VV2D and VV3D, with ICCs ≥0.98 (Table 1). There was no significant difference between the means of M1 and M2 for any angles. Absolute intraobserver reliability showed no significant bias between for VV2D and VV3D. The SEM was 0.20° and the SDC 0.55° for VV2D. For VV3D, the SEM was 0.43° and the SDC 1.20°. The calculated κ coefficient was 0.94 for both VV2D and VV3D.

**Table:** **Table 1.** Reported intraobserver and interobserver reliability of varus/valgus angle measured in 2D and 3D.

Intraobserver reliability									
	M1*	M2*	Difference M1–M2 (95% CI)	Range of differenceM1–M2	P-value	ICC (95% CI)	SDΔ	SEM	SDC
VV2D	−1.0 (3.7)	−1.0 (3.8)	−0.02 (−0.11, 0.07)	−0.7, 0.6	0.66	1.00 (1.00, 1.00)	0.28	0.20	0.55
VV3D	−1.9 (3.2)	−2.0 (3.4)	0.08 (−0.11, 0.28)	−1.3, 2.7	0.39	0.98 (0.97, 0.99)	0.61	0.43	1.20
Interobserver reliability									
	Obs. A*	Obs. B*	Difference Obs. A–B (95% CI)	Range of difference obs. A–B	P-value	ICC 95% CI	SDΔ	SEM	SDC
VV2D	−1.1 (3.8)	−0.9 (3.7)	−0.16 (−0.34, 0.03)	−1.5, 0.8	0.85	0.99 (0.98, 0.99)	0.58	0.41	1.14
VV3D	−2.0 (3.4)	−1.9 (3.2)	−0.10 (−0.39, 0.19)	−2.5, 2.2	0.90	0.96 (0.93, 0.98)	0.91	0.64	1.77

*Results are given as mean (sd). Abbreviations: VV2D: varus/valgus angle in 2D, VV3D: varus/valgus angle in 3D, M1: measurement session 1, M2: measurement session 2, sd: standard deviation, CI: confidence interval, ICC: intraclass correlation coefficient, SDΔ: standard error of difference, SEM: standard error of measurement, SDC: smallest detectable change, Obs: observer. Angles are expressed in degrees (°).

Relative interobserver reliability was excellent for both angles, with ICCs ≥0.96 (Table 1). There was no significant difference between the measurements of observer A and observer B. Absolute interobserver reliability of VV2D and VV3D showed no significant bias between the measurements of the two observers. The SEM was 0.41° and the SDC 1.14° for VV2D. For VV3D, the SEM was 0.64° with an SDC of 1.77°. The κ coefficient was 0.78 for VV2D and 0.88 for VV3D.

There was a significant mean and absolute difference between VV2D and VV3D measurements. The mean difference between VV2D and VV3D was 1.00° (1.66–0.34) (p = 0.004) and the mean absolute difference was 1.61° (1.09–2.13), with a p-value of <0.001 (Table 2). The κ coefficient for the agreement between the outliers as determined on 2D and 3D was 0.50.

**Table:** **Table 2.** Difference between 2D and 3D varus/valgus angle measurements.

	Mean	SD	Mean difference	95% CI	P-value
VV2D	−.98	3.75	1.00	1.66–0.34	0.004
VV3D	−1.97	3.28			
Diff	1.61	1.62		1.09–2.13	<0.001

Abbreviations: VV2D: varus/valgus angle in 2D, VV3D: varus/valgus angle in 3D, sd: standard deviation, CI: confidence interval, SDΔ: standard error of difference, SEM: standard error of measurement, SDC: smallest detectable change. Results are given as mean (sd). Angles are expressed in degrees (°).

Scatter graphs of the Bland & Altman method are presented in Figure S1, Figure S2, Figure S3, and Figure S4. Tables of the distribution of outliers are reported in Table S1.

## Discussion

A new low-dose X-ray device, called EOS, was recently introduced for determining lower-limb alignment in 2D and 3D [7]. Reliability has not yet been assessed when performing EOS measurements on lower limbs containing a knee prosthesis. Purpose of this study was to determine intraobserver and interobserver reliability of 2D and 3D knee prosthesis alignment measurements after rTKA. Potential differences between 2D and 3D measurements were assessed as a secondary outcome.

Intraobserver and interobserver reliability were excellent for VV2D and VV3D, with no significant differences or systematic bias between the measurements of the two measurement sessions or observers. SEM and SDC of both VV2D and VV3D were small, but larger for VV3D. The κ coefficients showed substantial to almost-perfect intraobserver and interobserver reliability for determining outliers, for both 2D and 3D measurements. A significant mean and absolute difference existed between the angles measured in 2D and 3D.

Results of this study are comparable to other studies investigating reliability of EOS. Intraobserver and interobserver reliability were excellent when measuring VV2D and VV3D (with ICCs >0.99) on lower limbs containing no knee prostheses [18]. Interobserver reliability was good for EOS 3D varus/valgus measurements on lower limbs of children containing no knee prostheses (Pearson correlation coefficient (Pr) 0.82) [19]. Reliability studies on measurements of vertebrae [20], sagittal balance and spine curves (Pr ≥0.85 and ICCs ≥0.85) [21], spinal curve measurements (ICCs ≥0.84) [22], scoliosis (ICCs ≥0.97) [23], shoulder bony landmarks [24], pelvic and acetabular morphology (ICCs ≥0.80) [25], and pelvic tilt and acetabular cup orientation (ICCs 0.69–0.98) [26], also showed good overall reliability.

SEM and SDC for VV3D were greater than VV2D for both intraobserver and interobserver reliability. A smaller SEM and SDC means that measurements are more precise, but that doesn't indicate which of the two measurement types is more accurate or valid. In this study the SEM and SDC were larger for VV3D than for VV2D. This can be explained by the way in which 2D and 3D measurements are calculated. Since a 3D measurement is calculated through a combination of two planes (coronal and sagittal) and a 2D measurement is measured in the coronal plane only, slightly more variation can be expected in the 3D measurements and thus a higher SEM and SDC.

One could debate whether the significant differences between 2D and 3D measurements are of clinical importance. Both the mean and absolute difference were small (1.00° (1.66–0.34) and 1.61° (1.09–2.13), respectively). The mean difference is smaller than the absolute difference. For the absolute difference, we stated the deviation of the neutral axis as a positive value, regardless of the deviation being varus or valgus. For the mean difference, varus was stated as a negative value and valgus as a positive value. Calculating the mean difference using both positive and negative values, the deviation may be underestimated. There was only a moderate agreement between 2D and 3D measurements for assessment of outliers — meaning that in 2D different lower limbs are defined as outliers than in 3D.

The influence of lower-limb positioning on 2D measurements has been shown in previous studies. Varus or valgus deformity, axial rotation and flexion of the lower limb at the time of assessment of the radiographs alter coronal measurements of knee alignment [27]–[30]. When a measurement is taken in 3D, the system mathematically corrects for potential malpositioning during acquisition. EOS VV3D measurements of legs that not contain a knee prosthesis are more accurate than VV2D measurements, eliminating bias due to wrong lower-limb positioning [31]. Validity of EOS VV3D on legs not containing prosthetic material was also investigated in a cadaveric study [19] that measured varus/valgus angle three times using CT-scanning and EOS 3D with each specimen in three different positions: neutral, 10° external rotation and 10° internal rotation. No significant differences between CT and EOS 3D measurements were observed. To gain more insight into validity, additional research has to be conducted in which the accuracy of VV2D and VV3D EOS measurements on lower limbs containing a knee prosthesis are investigated.

This study has some limitations. First of all, when generating a 3D reconstruction of the lower limb with the EOS software it is possible to use the full 3D mode or the lower-limb alignment mode. When using the full 3D mode more angles can be calculated for knee prosthesis alignment, but even more landmarks that have disappeared or changed have to be identified. Hence it was decided not to use the full 3D mode because of a greater chance of errors. Secondly, when it was not possible to identify the medial and lateral condyles on the lateral images, the patient was excluded. In order to identify them on both the AP and the LAT image the condyles have to differ in height on the EOS images. To prevent this in the future, whether the condyles differ in height has to be checked directly after acquisition, otherwise acquisition has to be repeated. Finally, no generally accepted measurement protocol exists for 3D reconstructions of limbs with knee prosthesis material *in situ*. To tackle this issue, the two observers drew up a measurement protocol.

Our study showed that EOS provides reliable varus/valgus measurements of lower limbs containing a revision knee prosthesis in 2D and 3D. There is however a significant difference between varus/valgus measurements in 2D and 3D.

## Supporting Information

Figure S1 Bland-Altman plot of intraobserver reliability for VV2D. The dotted line represents the mean difference and the dark lines represent the borders of the 95% confidence intervals.
(TIF)

Figure S2 Bland-Altman plot of intraobserver reliability for VV3D.
(TIF)

Figure S3 Bland-Altman plot of interobserver reliability for VV2D.
(TIF)

Figure S4 Bland-Altman plot of interobserver reliability for VV3D.
(TIF)

Table S1 Tables of the distribution of outliers.
(DOC)
