# Supplementary figures and images for: Assessment of Prosthesis Alignment after Revision Total Knee Arthroplasty Using EOS 2D and 3D Imaging: A Reliability Study
Source: PLoS One. 2014 Sep 23;9(9):e104613. doi: 10.1371/journal.pone.0104613 (PMC4172435; doi:10.1371/journal.pone.0104613)

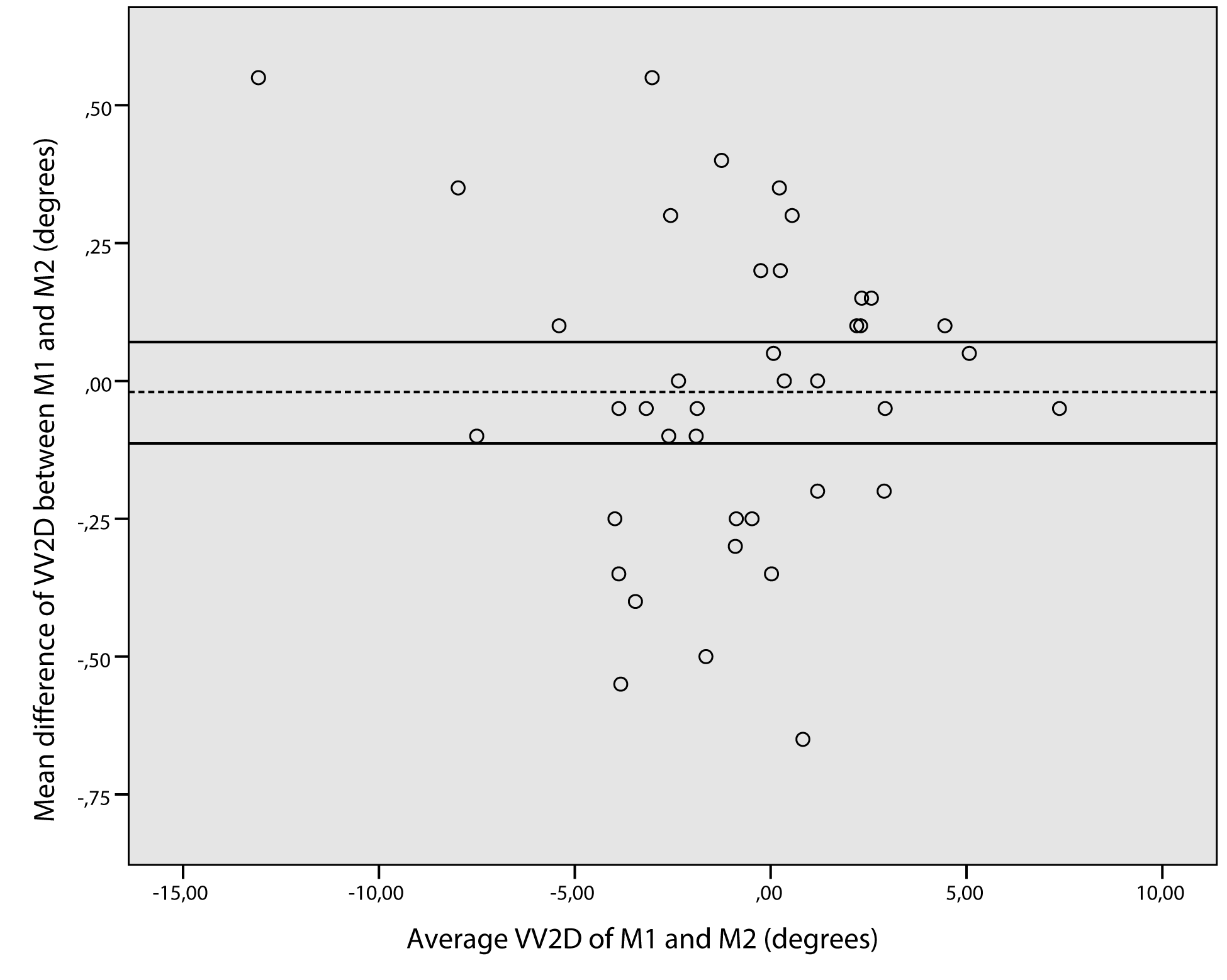

Supplement: Figure S1 — Bland-Altman plot of intraobserver reliability for VV2D. The dotted line represents the mean difference and the dark lines represent the borders of the 95% confidence intervals. (TIF) [file pone.0104613.s001.tif]

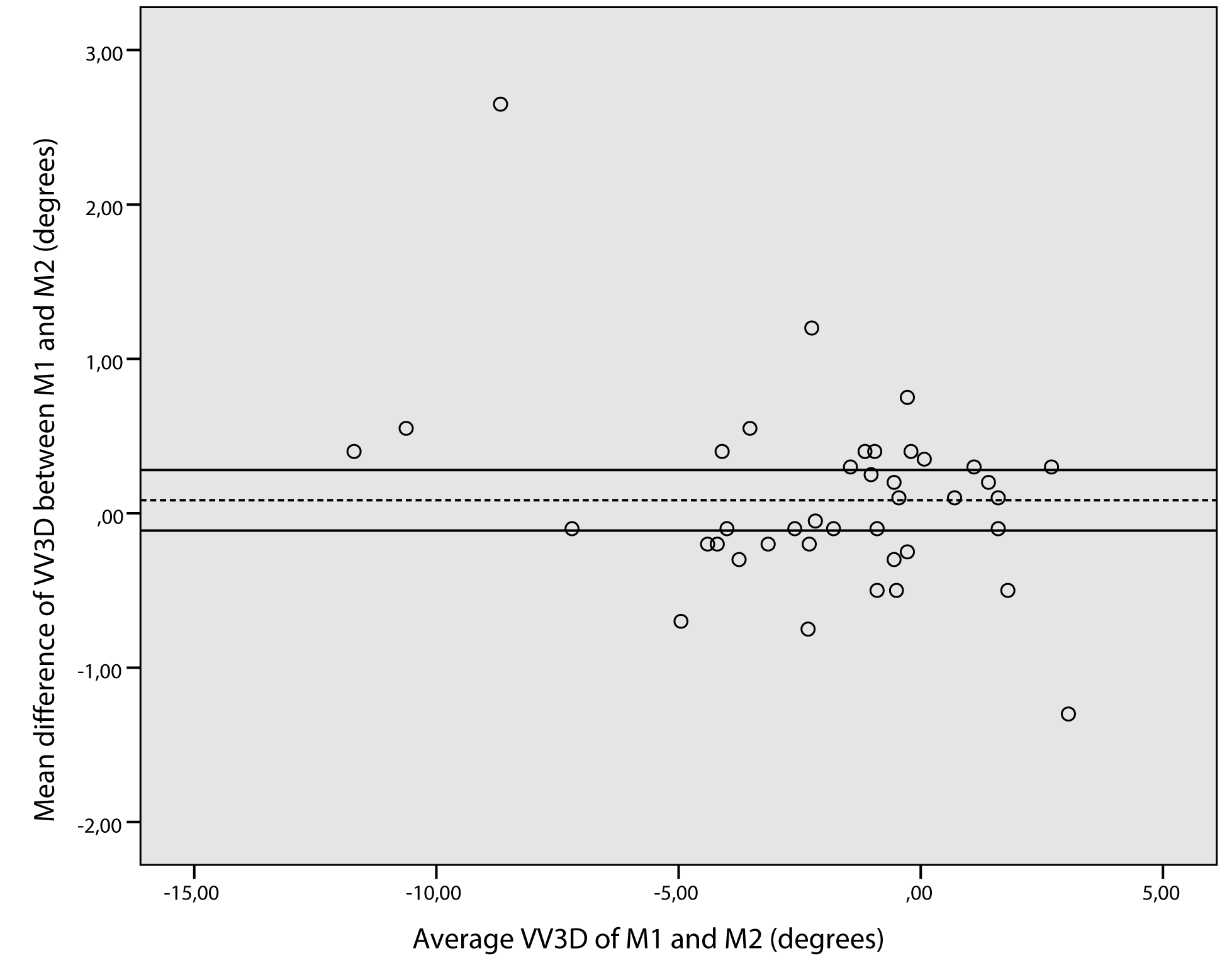

Supplement: Figure S2 — Bland-Altman plot of intraobserver reliability for VV3D. (TIF) [file pone.0104613.s002.tif]

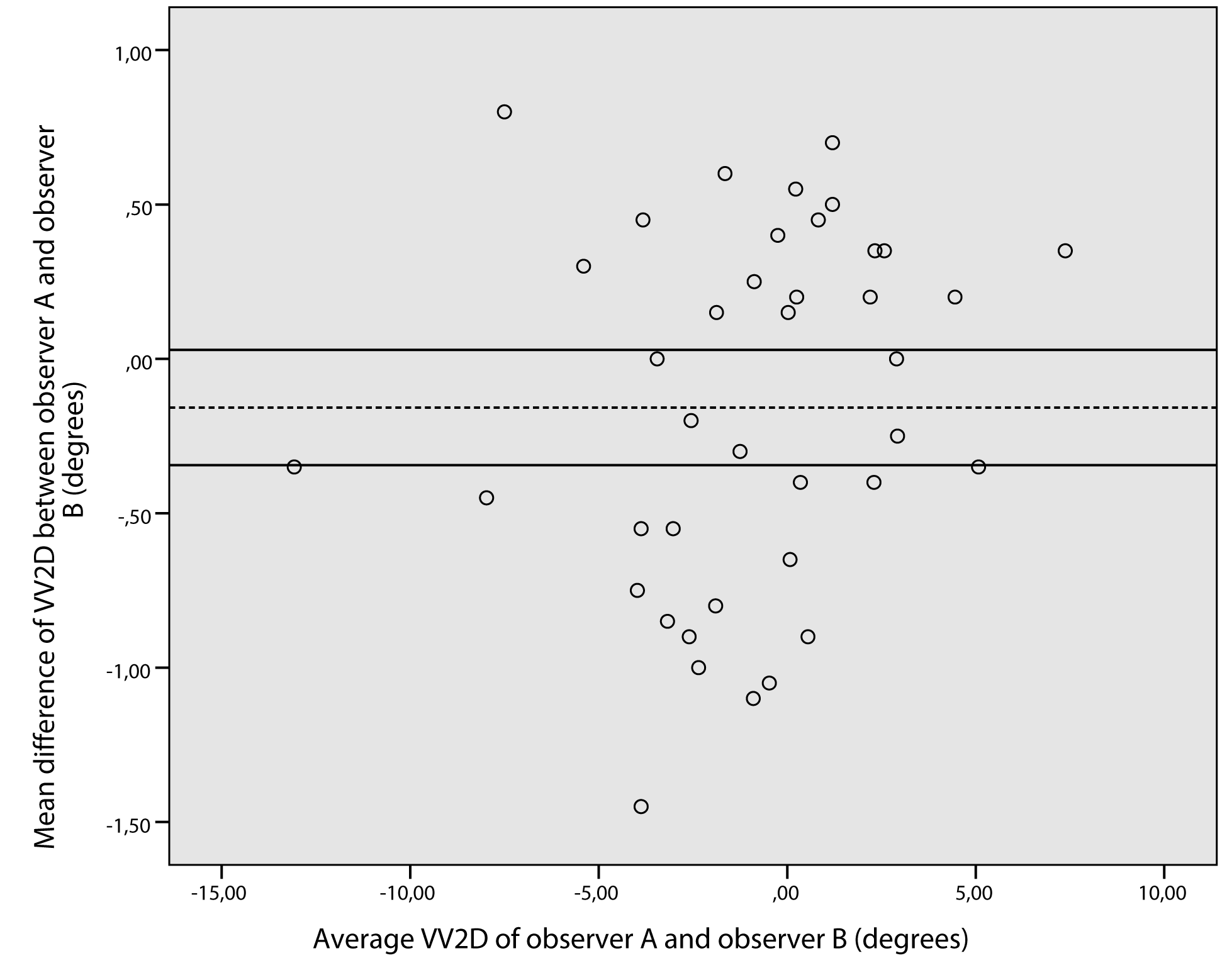

Supplement: Figure S3 — Bland-Altman plot of interobserver reliability for VV2D. (TIF) [file pone.0104613.s003.tif]

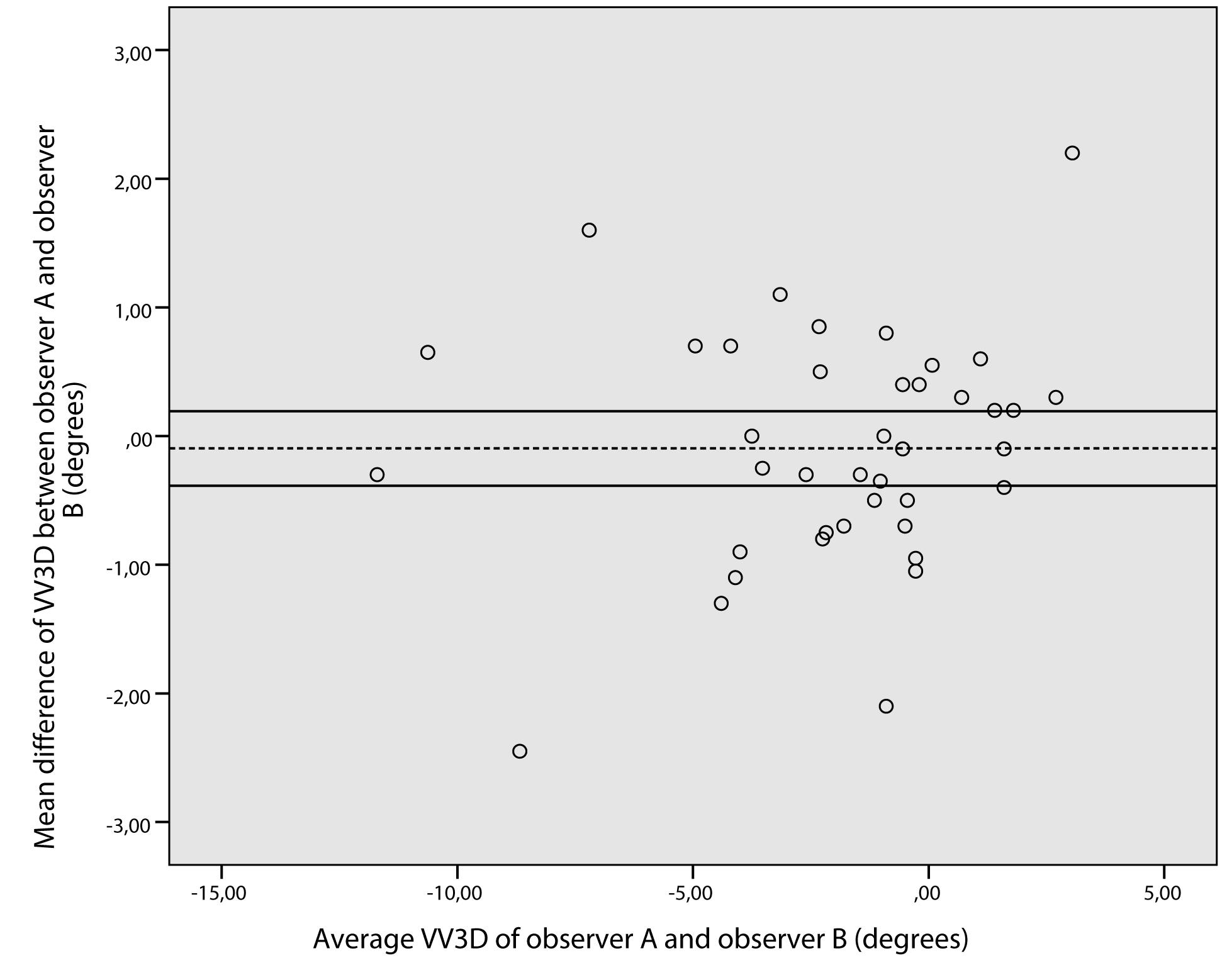

Supplement: Figure S4 — Bland-Altman plot of interobserver reliability for VV3D. (TIF) [file pone.0104613.s004.tif]
