# Supplementary material for: Assessment of Prosthesis Alignment after Revision Total Knee Arthroplasty Using EOS 2D and 3D Imaging: A Reliability Study
Source: PLoS One. 2014 Sep 23;9(9):e104613. doi: 10.1371/journal.pone.0104613 (PMC4172435; doi:10.1371/journal.pone.0104613)
Supplement: Table S1 — Tables of the distribution of outliers. (DOC) [file pone.0104613.s005.doc]

| Outlier distribution of intraobserver reliability for VV2D | | | | |
| --- | --- | --- | --- | --- |
|  | | | | |
|  | | VV2D M2 | | Total |
|  |  | No outlier | Outlier |  |
| VV2D M1 | No outlier | 26 | 1 | 27 |
|  | Outlier | 0 | 13 | 13 |
| Total | | 26 | 14 | 40 |

| Outlier distribution of intraobserver reliability for VV3D | | | | |
| --- | --- | --- | --- | --- |
|  | | | | |
|  | | VV3D M2 | | Total |
|  |  | No outlier | Outlier |  |
| VV3D M1 | No outlier | 27 | 1 | 28 |
|  | Outlier | 0 | 12 | 12 |
| Total | | 27 | 13 | 40 |

| Outlier distribution of interobserver reliability for VV2D | | | | |
| --- | --- | --- | --- | --- |
|  | | | | |
|  | | VV2D Observer B | | Total |
|  |  | No outlier | Outlier |  |
| VV2D Observer A | No outlier | 24 | 1 | 25 |
|  | Outlier | 3 | 12 | 15 |
| Total | | 27 | 13 | 40 |

| Outlier distribution of interobserver reliability for VV3D | | | | |
| --- | --- | --- | --- | --- |
|  | | | | |
|  | | VV3D Observer B | | Total |
|  |  | No outlier | Outlier |  |
| VV3D Observer A | No outlier | 27 | 1 | 28 |
|  | Outlier | 1 | 11 | 12 |
| Total | | 28 | 12 | 40 |

| Outlier distribution of VV2D versus VV3D | | | | |
| --- | --- | --- | --- | --- |
|  | | | | |
|  | | VV2D | | Total |
|  |  | No outlier | Outlier |  |
| VV3D | No outlier | 22 | 5 | 27 |
|  | Outlier | 4 | 9 | 13 |
| Total | | 26 | 14 | 40 |
